# New Appliances and Things Medical

**Published:** 1903-10-17

**Authors:** 


### NEW APPLIANCES AND THINGS MEDICAL.

[We shall be glad to receive at our Office, 28 & 29 Southampton Street, Strand, London, W.C., from the manufacturers, specimens of all new preparations and appliances which may be brought out from time to time.]

### MYKROL.

(The Bone Phosphate and Chemical Company, Limited, Flint, North Wales.)

A new disinfectant preparation of this name has been lately placed on the market. It is a dark brown liquid, forming with water an opalescent solution with a not unpleasant odour, and has the important property of being non-poisonous. According to an authoritative analytical report, its germicidal powers are powerful, for it is therein stated that a 1 per cent. solution will destroy the bacillus of enteric fever in five minutes, while a 2 per cent. solution will destroy the anthrax bacillus and the bacillus coli communis in the same time. This disinfectant should prove a useful addition to the already long list of these preparations.

### STRENBO.

(Moseleys Food, Limited, Stockport.)

The manufacture of this extract of meat has been recently undertaken by the well-known firm "Moseleys Food, Limited," in whose hands its excellence will remain unimpaired. There are few preparations which mislead the public, and even many members of the medical profession, more than beef extracts, so that it becomes the more important to lay stress on such an extract as "Strenbo, which is essentially a food as well as a stimulant. It contains a high percentage of soluble and assimilable proteid and possesses a pronounced palatability—a most necessary quality in the case of invalids. We can heartily commend "Strenbo" as a valuable and reliable meat extract.

